# Torque teno viral load reflects immunosuppression in paediatric kidney-transplanted patients—a pilot study

**DOI:** 10.1007/s00467-020-04606-3

**Published:** 2020-06-10

**Authors:** Phoebe Uhl, Andreas Heilos, Gregor Bond, Elias Meyer, Michael Böhm, Elisabeth Puchhammer-Stöckl, Klaus Arbeiter, Thomas Müller-Sacherer, Dagmar Csaicsich, Christoph Aufricht, Krisztina Rusai

**Affiliations:** 1 Department of Paediatrics and Adolescent Medicine, Comprehensive Center for Pediatrics, Division of Paediatric Nephrology and Gastroenterology, Medical University of Vienna, Vienna, Austria; 2 Department of Medicine III, Division of Nephrology and Dialysis, Medical University of Vienna, Vienna, Austria; 3 Center for Medical Statistics, Informatics and Intelligent Systems, Medical University of Vienna, Vienna, Austria; 4 Department of Virology, Medical University of Vienna, Vienna, Austria

**Keywords:** Anellovirus, Torque teno virus, TTV, Transplantation, Paediatric kidney transplantation, Immunosuppression, Immunologic monitoring

## Abstract

**Background:**

Chronic deterioration of kidney graft function is related to inadequate immunosuppression (IS). A novel tool to assess the individual net state of IS in transplanted patients might be the monitoring of Torque teno virus (TTV) viral load. TTV is a non-pathogen virus detectable in almost all individuals. TTV level in the peripheral blood has been linked to the immune-competence of its host and should thus reflect IS after solid organ transplantation.

**Methods:**

TTV plasma load was quantified monthly by RT-PCR for a period of 1 year in 45 kidney-transplanted children. Post-transplant time was at least 3 months. The relation of the virus DNA levels to IS and transplant-specific clinical and laboratory parameters was analysed longitudinally.

**Results:**

TTV DNA was detectable in 94.5% of the plasma samples. There was a significant association with the post-transplant follow-up time as well as with the type of IS regimen, with lower virus loads in patients after longer post-transplant time and mTOR inhibitor–based IS. Furthermore, a significant positive correlation with the dose of prednisolone and mycophenolate mofetil was found.

**Conclusions:**

TTV levels show an association/correlation with the strength of IS. Further studies are needed in order to evaluate TTV measurement as a tool for IS monitoring for hard clinical outcomes such as presence of donor-specific antibodies, rejections or infections—common consequences of insufficient or too intense IS.

**Electronic supplementary material:**

The online version of this article (10.1007/s00467-020-04606-3) contains supplementary material, which is available to authorized users.

## Introduction

Immunosuppression (IS)-related adverse events are still the main factors limiting long-term transplant survival [1]. Intense IS leads to deleterious medication side effects, and to increased rates of opportunistic infections, with higher risk of direct infectious complications, malignancies and allograft damage [1–3]. On the other hand, sustained insufficient IS can lead to inferior graft outcome primarily due to activation of the humoral alloimmune response with development of de novo anti-HLA donor-specific antibodies (DSA), which can cause late antibody-mediated rejection (AMR) [1, 4, 5].

In order to prevent IS-associated complications, reliable and feasible tools for individual IS monitoring must be introduced into the clinical setting.

Currently, monitoring of IS is still mainly based on the measurement of calcineurin inhibitor (CNI) or mTOR inhibitor (mTORi) trough levels. However, drug blood levels do not reveal the net effect of the combined IS therapy, nor individual differences or, more importantly, the real functional immune status [6]. Thus, there is still a need for an applicable, non-invasive tool to better guide and tailor the IS therapy.

A promising and feasible method to estimate the status of IS in transplanted patients might be the assessment of the Torque teno virus (TTV) plasma load. TTV is a non-enveloped, negative-sense, single-stranded, circular DNA virus belonging to the Anelloviridae family [7], which has recently been shown to represent a large fraction of the human respiratory [8] and blood virome [9, 10].

Commonly acquired very early in life [11], the virus usually leads to a persistent, possible lifelong low-level viremia [12] in up to 90% [13] of the population. It has been shown to reside in peripheral blood mononuclear cells, as well as in various other tissues such as the respiratory epithelium, liver cells and the thyroid gland [14, 15], and seems to be ubiquitous in humans. Other characteristics are that the virion does not exhibit seasonal fluctuations or epidemic spikes [16], is insensitive to the antiviral drugs used in solid organ transplant (SOT) recipients [9, 17] and can be assayed using rapid molecular techniques [11, 18]. Moreover, to date, despite extensive research, no specific human illness has been casually linked to TTV currently classified as a disease orphan virus [7].

Therefore, TTV has gained growing attention as a potential marker of the functional immune status over the last years. TTV load in blood was shown to be significantly elevated in immunocompromised individuals, such as HIV and cancer patients [19, 20], suggesting a relevant immune control of TTV in the healthy individual. Later on, several studies have investigated the dynamics of the TTV viral load in transplanted (lung, liver, heart and kidney) patients and could demonstrate an increase in the TTV copy number after establishment of the IS medication [21, 22]. Moreover, a correlation with the intensity of the IS could be demonstrated [23–25], with association between the viral load and the likelihood of IS-related complications such as infections and rejections [9, 17, 26–32]. Altogether, these data have suggested that the dynamic of the TTV plasma load (kinetic) could serve as a diagnostic and prognostic marker for IS in transplanted patients.

Since there are no data on TTV in kidney-transplanted children, the aim of this study was to investigate TTV load and its changes in a prevalent cohort of paediatric kidney-transplanted patients. Associations and correlations with transplantation-specific clinical and laboratory parameters were investigated, in particular IS medication and signs of insufficient or too intense IS.

## Methods

In the present study, all 60 prevalent paediatric kidney transplant recipients followed at the Department of Paediatrics and Adolescent Medicine, Medical University, Vienna, Austria, between November 2014 and December 2015 were included.

Four patients with a post-transplant time less than 3 months were excluded. This was defined based on existing literature describing the establishment of a steady state in TTV viral load approximately 3 months after transplantation [33, 34]. Patients with fewer than seven TTV measurements were also excluded (*n* = 11). Demographics and characteristics of the remaining 45 study patients are outlined in Table 1.

**Table 1:** Main characteristics of the study patients (*CAKUT*, congenital anomalies of the kidney and urinary tract; *CKD*, chronic kidney disease; *DDKD*, deceased donor kidney transplantation; *DSA*, donor-specific antibodies; *IQR*, interquartile range; *LDKD*, living donor kidney transplantation; *n*, patient number; *SD*, standard deviation; *y*, years)

Demographics of the study patients	
Number of patients	45
Recipient data	
Age: mean (SD), in years (y)	12.8 (**±** 5.2)
Gender (male/female): *n* (%)	31 (69%)/14 (31%)
Age at transplantation: median (range), in years (y)	8.3 (17.6)
Post-transplantation time: mean (SD) in years (y)	4.5 (± 4.2)
Kidney disease leading to CKD stage 5: *n* (%)*	
CAKUT	23 (51.1%)
Glomerular disease	5 (11.1%)
Cystic kidney disease	8 (17.8%)
Nephrotic syndrome	6 (13.3%)
Metabolic disease	2 (4.4%)
Transplant data	
Donation type (DDKT/LDKT): *n* (%)	15 (33%)/30 (67%)
HLA mismatch: *median (IQR)*	3 (2–3)**
DSA+/DSA− at study begin: *n (%)*	8 (18%)/37 (82%)
Immunosuppression at study begin: ***n*** **(%)**	
Tacrolimus	39 (86.7%)
Cyclosporin	3 (6.7%)
Rapamycin	3 (6.7%)
Mycophenolate mofetil	38 (84.4%)
Azathioprine	4 (8.9%)
No anti-proliferative agent	3 (6.7%)
Corticosteroid	45 (100%)

*Missing data to one patient

**Missing data to 4 patients

Prior to transplantation, all study patients had a negative complement-dependent lymphocytotoxicity crossmatch. IS treatment protocol consists of corticosteroids, mycophenolate mofetil or azathioprine, and calcineurin inhibitors (CNI, tacrolimus or cyclosporin) or rapamycin. In detail, 39 patients were treated with tacrolimus, 3 patients with cyclosporin and 3 patients with rapamycin. 35 patients received mycophenolate mofetil and 4 patients azathioprine (triple IS). Patients without anti-metabolites (6 patients) were defined as patients with dual IS.

The ethics committee of the Medical University of Vienna approved the protocol (EK Nr. 1255/2015), and the study was performed in accordance with the Declaration of Helsinki.

### Study design

Patients were regularly monitored for plasma TTV DNA load for a period of 1 year, on average once monthly as part of the routine follow-up visits.

Clinical data, including gender, age, post-transplant time, primary disease, type of donation, number of mismatches and IS medication, were collected for all patients from their medical records.

Laboratory data such as estimated glomerular filtration rate (eGFR), medication trough levels (tacrolimus, cyclosporin, rapamycin), CMV, EBV and BKV plasma load were documented monthly. Doses of IS medication as well as non-adherence and events of infections or rejections were also recorded. Patients were screened for the presence of DSA at the beginning of the study period.

Association of clinical data with the mean TTV load during the 12-month period was analysed. Correlation of the TTV values measured at each time point with the eGFR, IS medication doses and trough levels, EBV, CMV and BKV plasma load assessed at the corresponding time points was analysed for each patient.

Association of the TTV values measured at each time point with infections and with the presence of non-adherence was investigated. Furthermore, association between TTV value measured 1 month before onset of infection and the presence of infections was also analysed.

Biopsy was not performed routinely, but only in patients with signs of graft dysfunction and scored following the 2013 update of the BANFF scheme.

### Laboratory and clinical parameters

The eGFR was calculated by the original Schwartz formula based on serum creatinine values [35].

Levels of EBV-, CMV-, and BKV-DNA in plasma were determined with virus-specific TaqMan real-time PCR assays [36, 37].

Medical records were screened for infectious episodes and suspicion of non-compliance. Infectious complications were defined as any bacterial, viral or fungal infection with or without fever. Urinary tract infections (UTIs) were excluded from the analysis since occurrence of UTIs in this patient population is rather a consequence of the underlying urological issues than of the IS alone [38]. Infectious complications with fever were further analysed as a subgroup population.

Non-compliance was recorded by routine multidisciplinary (at least every 3rd month) patient/parents consulting through clinicians and psychologists.

DSA was assessed by a Luminex-based bead array assay (LABScreen™ Mixed, One Lambda, Canoga Park, CA, USA) as described previously [5].

### TTV DNA Quantitation

TTV DNA extraction, purification and quantitation were performed as previously described [26, 39, 40]. Presence and load of TTV were determined by a universal TaqMan RT-PCR assay targeted to a highly conserved segment of the noncoding region of the viral genome. The procedures used for quantification of copy numbers and evaluation of specificity, sensitivity, intra- and inter-assay precision, and reproducibility of the assay have been described elsewhere [26, 39, 40]. Results were recorded in log10 copies/ml. TTV DNA quantitation was in the linear range from 2 to 10 log10 copies/ml as determined by the use of 10-fold dilutions of a plasmid standard. The limit of detection was 2 log10 copies/ml of plasma.

### Statistics

Categorical data are presented as absolute numbers and percentages, while summaries for continuous variables are given as median (interquartile range) or mean (+/− standard deviation). For all analyses, the total TTV-, EBV-, CMV- and BKV-DNA counts were log10 transformed to address the problem of huge relative differences and skewed distributions. Since no values in (0,1) are possible and no values between 1 and 20 occurred, values of 0 were set to 1 before transformation.

For analysing the effect of patient-specific characteristics on the TTV DNA plasma load, the mean TTV DNA plasma load across all time points was computed for every patient. Subsequently, ANOVA tests (categorical variables) and linear regression (continuous variables) were used.

Furthermore, in order to correlate TTV values and eGFR, IS medication doses and trough levels, EBV, CMV and BKV plasma load, Spearman’s rank correlation coefficient across all time points was computed for every patient. The mean correlation across all patients was computed and significance was tested using a 10,000 iteration permutation test.

To investigate the effect of TTV load on the likelihood of infectious episodes and non-adherence, mixed model logistic regressions were used. Data from all time points was used and four models were fitted, always including a patient intercept as random effect and the TTV load as fixed effect, with the four binary outcome variables being infectious episode (microbial or febrile) or non-adherence at the same time point and microbial infectious episode at the subsequent time point.

Due to the exploratory nature of the study, no adjustments for multiple testing were made and the calculated *p* values serve only descriptive and hypothesis-generating purposes. MS Excel 2016 (Redmon, WA) as well as R, version 3.3.2 (R core team, 2016, R Foundation for Statistical Computing, Vienna, Austria; http://www.R-project.org/), were used for data management, analysis and plotting of data.

## Results

### Characteristics of the study population

Serial samples for TTV quantification were obtained from 45 paediatric kidney allograft recipients at a median post-transplant follow-up time of 4.6 years. Clinical characteristics of the study patients are listed in Table 1. Briefly, male/female ratio was 69%/31% and the kidney allograft was more likely from a living donor (67%) than from a deceased donor (33%). The mean age of the cohort at study initiation was 12.8 years. The most common indication for kidney transplantation was an underlying congenital anomaly of the kidney or urinary tract (51%), followed by cystic kidney disease and nephrotic syndrome, affecting 18% and 13% of the patients, respectively.

### TTV DNA prevalence and viral load kinetics

All study patients had TTV infection. Virus genome was measurable at each time point of viral assessment in 40 patients. In three patients, the TTV DNA load was below the level of quantification for at least one measurement, and two patients had only one positive plasma sample and stayed TTV-negative throughout the 12-month study period.

In total, 509 plasma samples were available for TTV DNA load assessment, with a mean of 11 specimens per patient. Virus DNA was detectable in 94.5% (481 of 509) of the plasma samples with a median viral load of 5.8 log10 copies/ml (IQR, 4.5–6.7 log10). Overall, the TTV copy number ranged between 2.34 and 10 log10/ml, showing a high interindividual variability.

### TTV plasma load in relation to patients’ clinical characteristics

**Fig. 2:**
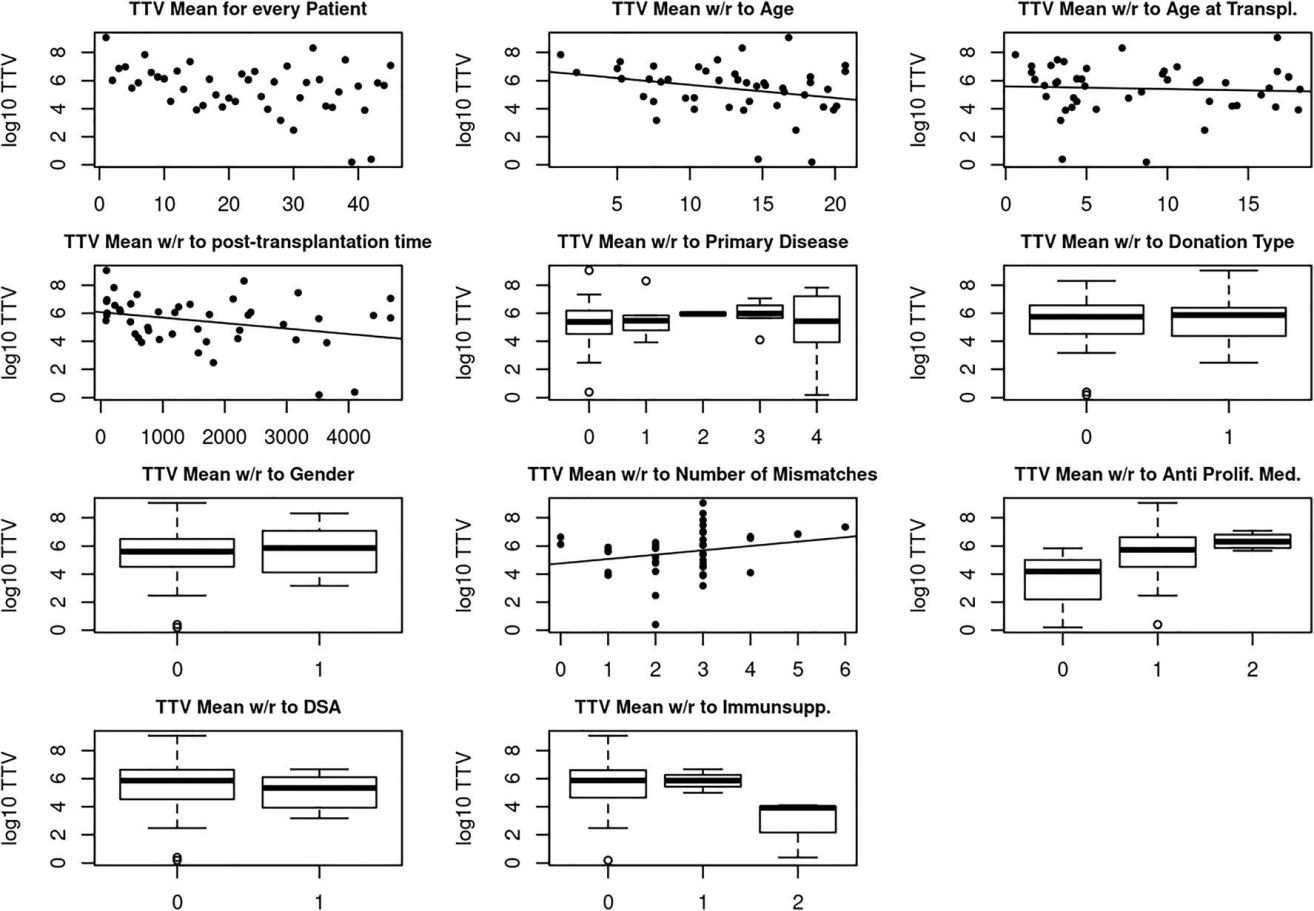
Association of the mean log10 TTV levels with the main patient characteristics (from left to right: TTV mean for every patients, association with age (years), age at transplantation (years), post-transplantation time (years), primary disease (0 = CAKUT, 1 = glomerular disease, 2 = cystic kidney disease 3 = nephrotic syndrome, 4 = metabolic disease), donation type (0 = living donor, 1 = diseased donor), gender (0 = male 1 = female), number of mismatches, anti-proliferative medication (0 = none, 1 = mycophenolate mofetil, 2 = azathioprine), donor-specific antibody (0 = no, 1 = yes), immunosuppression prescription (0 = tacrolimus, 1 = cyclosporine A, 2 = rapamycin)). Boxplots were drawn regardless of group sample sizes (for group sample sizes, see Table 1)

In order to detect possible differences in mean TTV load with respect to clinical variables, associations between gender, age, age at transplantation, post-transplant time, primary disease, type of donation, HLA mismatch and presence of DSA with the mean TTV plasma load of the 12-month study period were analysed (Table 2) (Fig. 2).

**Table 2:** Association of patients’ clinical parameters with the mean log10 TTV levels over the 12-month study period (*CNI*, calcineurin inhibitors; *GFR*, glomerular filtration rate; *mTORi*, mammalian target of rapamycin inhibitors, *m*^*2*^, square meters)

Mean log10 TTV during the 12-month study period	
Baseline parameters	*p* value
Gender	0.420
Primary disease	0.917
Age at transplantation	0.069
Type of donation	0.722
Number of mismatches	0.145
Follow-up parameters	
Age at study begin	0.695
Post-transplantation time	0.041*
Presence of donor-specific antibodies (DSA)	0.517
Immunosuppressive therapy	
Triple or dual immunosuppression (with or without anti prolif. medication)	0.076
Type of immunosuppression (CNI or mTORi-based)	0.023*

*p < 0.05

**Fig. 1:**
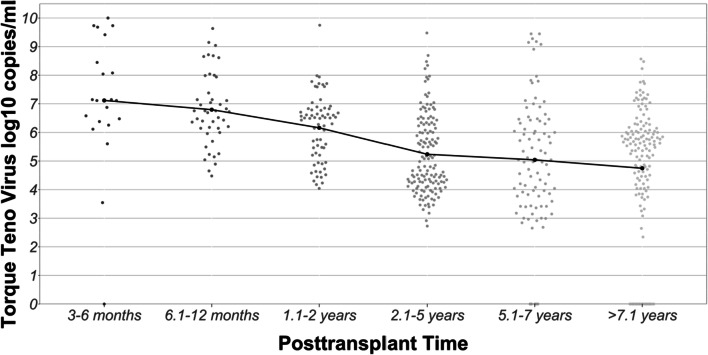
Violin scatterplot illustrating the log-transformed TTV viral load in peripheral blood of pediatric patients in relation to time after renal allograft transplantation. The mean viral loads for each post-transplant time are indicated as black dots and connected with a solid line

There was a significant association between the mean TTV load and the post-transplant time (*p* = 0.041). Patients with a shorter post-transplant time had higher viral loads than patients after several post-transplant years. TTV plasma loads according to the post-transplant time are shown in Fig. 1.

Furthermore, when analysing the association between recipient age and TTV plasma load, the *p* value was almost significant (*p* = 0.069). Patients below the age of five showed the highest viral load (analysis not shown). We found no significant association between the mean plasma viral load and age at transplantation, gender, type of donation or number of mismatches (all *p* values > 0.05).

### TTV plasma load in relation to the IS therapy

Analysis of the various maintenance IS treatment schemes (tacrolimus, cyclosporin or rapamycin, Table 2) (Fig. 2) suggested an association with the mean 12-month TTV load (*p* = 0.023). Patients with mTORi-based IS (rapamycin) had nearly 2 log10 lower mean TTV levels than patients who received one of the two CNI medications (tacrolimus or cyclosporin).

Similarly, patients on triple IS tended to have higher mean viral loads compared with patients who received no anti-metabolites (dual IS); however, this did not reach the level of significance (*p* = 0.069).

### Correlation of plasma TTV load with IS medication doses and laboratory parameters

Next, we investigated for each individual patient the correlation between the monthly TTV log10 load and the respective metric markers taken at the same follow-up visit. Mean correlation coefficients were calculated for all patients. The results of the bivariate analyses are summarised in Table 3.

**Table 3:** Correlation of the log10 TTV plasma load with the metric laboratory parameters and medication dosages over the course of the 12-month study period. (*BKV*, BK polyomavirus; *CMV*, human cytomegalovirus; *EBV*, Epstein-Barr virus; *GFR*, glomerular filtration rate)

	*p* value	Mean correlation coefficient
Immunosuppressive therapy		
Prednisolone/m^2^ dose	< 0.001*	0.224
Tacrolimus/kg dose	0.098	0.079
Cyclosporin/kg dose	0.282	0.242
Rapamycin/kg dose	0.056	0.356
Mycophenolate mofetil/m^2^ dose	0.011*	0.134
Azathioprine/kg dose	0.139	0.225
Tacrolimus trough level	0.110	0.080
Cyclosporin trough level	0.133	0.338
Rapamycin trough level	0.664	0.092
Virology		
EBV plasma load	0.950	0.002
CMV plasma load	0.986	− 0.001
BKV plasma load	0.060	0.049
eGFR	0.081	0.053

*p < 0.05

TTV load positively correlated with the dose of prednisolone (*p* < 0.001) as well as with the dose of mycophenolate mofetil (*p* = 0.011). Correlation with the dose of tacrolimus (*p* = 0.098) and rapamycin (*p* = 0.056) was almost significant.

There was no significant correlation between TTV plasma load and measured IS trough levels (tacrolimus/rapamycin/cyclosporin) (all *p* values > 0.05).

Analysing viral load data and graft function, correlation between TTV copy number and eGFR, BKV plasma load was almost significant (*p* = 0.081 and *p* = 0.060, respectively) (*p* = 0.081). Our remaining results did not indicate a correlation with any of the other measured viral loads (EBV and CMV in plasma) (all *p* values > 0.05).

### Relation of TTV load to infectious complications, non-adherence, DSA and rejections

According to our predefined criteria, 40 patients developed an infection at least once during the study period. In total, 119 infectious episodes occurred around the time point of a TTV measurement. Twenty of these cases were febrile infections. For this subset, a separate analysis was performed.

For all analyses, TTV load at the time of infection and TTV load at the month before the onset of infection were investigated. Altogether, we could not find a significant association between the TTV load at the time of, or a month before, onset of the microbial infection (*p* = 0*.*832 and *p* = 0*.*359, respectively). Furthermore, the subgroup analysis of the febrile infections revealed no significant association with the TTV copy number either (*p* = 0*.*584).

According to the patients’ files, eleven of the study participants (24%) were suspected to be non-adherent to their medication. As shown in Table 4, the odds ratio (OR) for non-adherence decreased by 11% per log10 level increase of TTV copies/ml, but this association did not reach the level of significance (95% confidence interval [CI], 0.691–1.154, *p* = 0.386).

**Table 4:** Mixed model logistic regression was used to analyse the relationship between the TTV viral load dynamic and respective outcome variables (*OR*, odds ratio)

	OR estimate	OR 2.5%	OR 97.5%	*p* value
Infection	1.016	0.876	1.180	0.832
Infection in the following month	1.075	0.921	1.25	0.359
Fever	0.932	0.724	1.1 99	0.584
Non-adherence	0.691	0.783	1.154	0.386

There was no association between the presence of DSA and TTV plasma load (analysis of association between DSA positivity and mean TTV load over the study period). Since none of the patients showed clinical or laboratory signs of graft dysfunction during the study period, association between TTV DNA level and the frequency of rejection episodes could not be analysed.

## Discussion

As long-term survival of the transplanted kidney is closely linked to various consequences of IS, different strategies are being explored to better guide the IS therapy. However, there is still an unmet need for a non-invasive tool to monitor and tailor the individual IS medication. Particularly, in the paediatric population, getting an insight into the individual status of IS is of high relevance due to multiple factors: (I) children have a very high morbidity risk regarding infections in the case of over-IS due to their immunological naivety prior to transplantation, (II) adolescents are more prone to under-IS due to non-adherence to their medication and (III) the young age demands ensuring a long-term graft survival.

Hence, the goal of our study was to explore the prevalent, non-pathogenic TTV as a potential marker of the functional immune status and to investigate its associations with various clinical and laboratory parameters in a yet unexplored cohort of kidney-transplanted children.

In accordance with previous data [9, 23, 24, 26, 33], our study revealed a high prevalence of TTV viremia and a relatively high TTV load in our cohort. The prevalence of TTV viremia measuring serial samples per patient was 94.5% with a median viral load of 5.8 log10 copies/ml. This is above the mean value of 2–4 log10 copies/ml commonly reported for healthy individuals [18, 27, 41], thus, showing an elevated viral burden.

Moreover, our values are consistent with previous results obtained from adult kidney [26, 30] and liver transplant recipients [23, 24, 27], while studies on lung allograft recipients have described higher viral loads of 7–10 log10 copies/ml in adults [33, 42] and 6–7 log10 copies/ml in children [28], respectively. The discrepancy in TTV copy number presumably reflects the different IS protocols applied depending on the transplanted organ [43, 44], different post-transplant periods [24, 26] and on the age of the transplant recipients [11, 24, 28, 45].

Our results revealed an association of the mean TTV load with the post-transplant time, showing that longer post-transplant time is associated with lower viral load. This is in line with a recent large cross-sectional study on adult kidney transplant recipients, which found the highest viral loads in patients screened after 6 to 12 months after transplantation and a stepwise decrease in levels at later time points [26]. Similarly, Béland et al. described a constant decline in the TTV plasma load up to 15 years post-transplantation in paediatric liver transplant recipients [24].

There are some studies which, in accordance with our results, have demonstrated an association between higher viral loads and the number of IS prescriptions administered in combination [23, 25, 26, 46]. Interestingly, Nordén et al. further reported that the type of CNI influenced the TTV viral burden, but not the EBV load, suggesting that TTV might be a more sensitive marker of the IS state than other viruses [34]. An interesting finding of our study was an association between mean TTV DNA level and IS medication: patients with rapamycin instead of CNI had significantly lower plasma levels of TTV. Moreover, patients on triple IS showed higher mean TTV viral loads; however, this association did not reach the level of significance. In line with our study, Schiemann et al. have also detected 2-fold higher TTV DNA levels for tacrolimus-based IS and the lowest viral load in adult kidney transplant recipients on mTORi-based protocols [26]. Altogether, these results also indicate that mean TTV viral load reflects the strength of IS in this cohort.

Our results analysing the correlation of TTV load with the dose of the different IS medications further confirmed that measured TTV plasma values might reflect the IS state. TTV positively correlated with the dose of prednisolone and MMF. The *p* value for correlation with tacrolimus and rapamycin dose was almost significant as well.

Only a few reports have investigated the TTV copy number in relation to the dose of the IS medications. Like in the current study, De Vlaminck et al. have demonstrated an increasing representation of anelloviruses, including TTV, with higher dose of prednisolone and tacrolimus [9]. Furthermore, Strassl et al. found an association between a higher copy number and the use of high-dose mycophenolic acid [17].

Results of studies investigating the association of the TTV viral load with the medication trough levels are inconsistent [8, 9, 17, 26, 33, 46, 47]. The majority of these reports have found no association [8, 17, 26, 46, 47], and our study also failed to detect a correlation between TTV load and tacrolimus, cyclosporin or rapamycin trough levels. The lack of correlation with serum drug levels confirms that measurement of trough levels itself might not reflect the net IS effect of the given medication and is not sufficient to guide its administration [6].

As we investigated the correlation of TTV with common transplant-specific viruses, we found no significant correlation; however, the *p* value was almost significant for BK virus. This result is in good accordance with the findings of Herrmann et al. [48] who described a positive correlation in liver-transplanted patients as well between the two viruses.

Analysing the graft function of the patients, TTV load negatively correlated with changes in eGFR. This could indirectly reflect the consequence of the deleterious side effects of the IS therapy [49], since high TTV load has been correlated with the dose of various IS medications. Another plausible explanation might be a possible correlation with recurrent post-transplant infections, which are known to contribute to allograft damage as well [50]. However, in the current study, we failed to demonstrate a significant association between the viral load and common infections.

As we analysed the relationship of TTV with non-adherence, a leading cause for insufficient IS, there was no significant association. Furthermore, there was no significant association with the presence of DSA, which commonly develop as a consequence of insufficient IS.

The current study has some limitations typical for a monocentric pilot trial, whereby the power of subgroup analysis was limited. Moreover, the incidence of infectious complications and non-adherence was low; therefore, the predictive value of the TTV viral load dynamic might have been underestimated.

It is important to highlight, however, that this is the first study in which the TTV DNA load has been assessed in paediatric kidney-transplanted patients. Furthermore, this is the first study in which TTV was measured in such short intervals after kidney transplantation (on average monthly) allowing a close monitoring of the viral load kinetic in correlation with clinical and laboratory parameters.

To summarise, TTV might serve as a parameter for the individual immune state helping to find the balance between over- and under-IS. It is important to note that a kind of steady state of the virus levels should be achieved in order to use TTV as a monitoring tool, which is approximately 3 months after transplantation. Some earlier studies have succeeded in defining certain cutoff levels for over- and under-IS rather than using intra-individual changes of TTV. For example, Görzer et al. [33] described a threshold level of 9.3 log10 copies/ml as predictive for development of infections (sign of too much IS) in lung transplant patients. Furthermore, Strassl et al. defined a TTV load between 6 and 8 log10 copies/ml as the optimal range to minimize the risk for rejection and infection in kidney transplant recipients [32]. Based on these results, an interventional trial assessing the efficacy of TTV-guided IS is being planned in this adult kidney transplant cohort.

In conclusion, our study is the first to investigate prospectively the course of TTV load and its association/correlation with various transplant-specific clinical and laboratory parameters in paediatric kidney transplant recipients. Based on our data, plasma TTV load seems to reflect the strength of IS in this cohort as medication prescription and changes of medication doses were analysed. Furthermore, TTV might correlate with BK virus load. The significance and relevance of this finding for the management of BK nephropathy need further evaluation.

Thus, to define TTV plasma load as a marker of the individual clinical IS state to guide IS therapy in this cohort, further and possibly multi-centre studies are needed with larger patient numbers.

## Electronic supplementary material

ESM 1 (DOCX 187 kb)

## Abbreviations

AMR: Antibody-mediated rejection
BKV: BK Polyomavirus
CAKUT: Congenital anomalies of the kidney and urinary tract
CNI: Calcineurin inhibitor
CMV: Cytomegalovirus
DDKD: Deceased donor kidney transplantation
DNA: Deoxyribonucleic acid
DSA: Donor-specific antibodies
EBV: Epstein-Barr virus
eGFR: Estimated glomerular filtration rate
HLA: Human leukocyte antigen
IS: Immunosuppression
JCV: JC Polyomavirus
LDKD: Living donor kidney transplantation
mTORi: Mammalian target of rapamycin inhibitors
MMF: Mycophenolate mofetil
post-Tx time: Post-transplantation time
SOT: Solid organ transplant
Tx: Transplantation
TTV: Torque teno virus
UTI: Urinary tract infection
