# Supplementary material for: Torque teno viral load reflects immunosuppression in paediatric kidney-transplanted patients—a pilot study
Source: Pediatr Nephrol. 2020 Jun 10;36(1):153–62. doi: 10.1007/s00467-020-04606-3 (PMC7701084; doi:10.1007/s00467-020-04606-3)
Supplement: Supplementary file 1 — (DOCX 187 kb) [file 467_2020_4606_MOESM1_ESM.docx]

Figure S1. Kinetics of torque teno virus (TTV)- DNA levels in plasma according to the post-transplantation time.


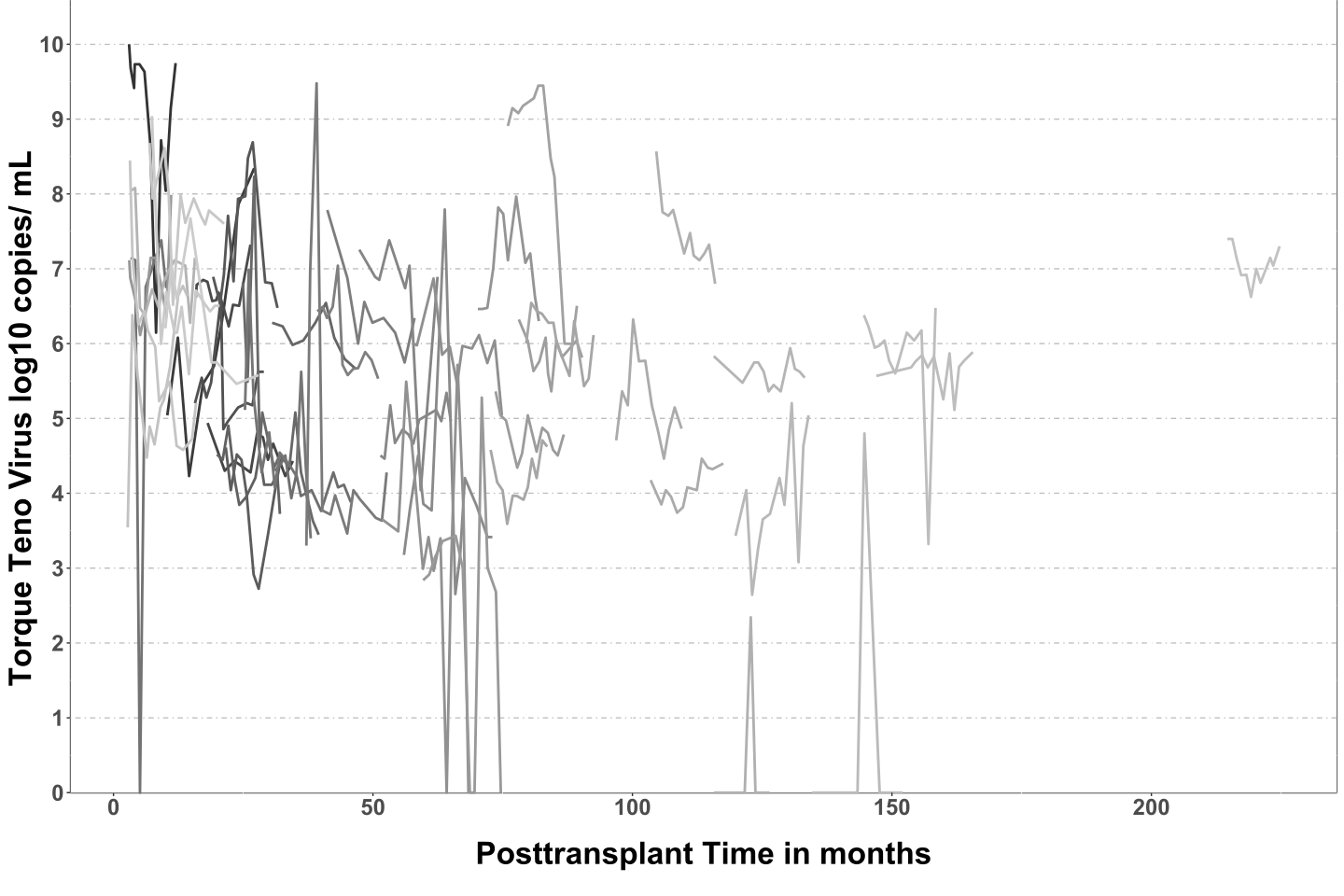


**Supplementary tables and figures**

Supplementary Table 1.

Number and types of infection for each patient during the study period.

| **Patients** | **Type and number of infections during the study period** |
| --- | --- |
| **1** | **none** |
| **2** | **none** |
| **3** | **none** |
| **4** | **none** |
| **5** | **2 episodes of febrile upper airway infection** |
| **6** | **1 episode of febrile upper airway infection** |
| **7** | **1 bacterial sepsis, three febrile upper airway infections** |
| **8** | **1 influenza infection, 3 episodes of non-febrile upper airway infection** |
| **9** | **none** |
| **10** | **three febrile upper airway infections, 1 mycoplasma pneumonia** |
| **11** | **1 episode of febrile upper airway infection** |
| **12** | **none** |
| **13** | **two episodes of febrile upper airway infection** |
| **14** | **five episodes of non-febrile upper airway infection** |
| **15** | **none** |
| **16** | **none** |
| **17** | **1 episode of non-febrile upper airway infection, 1 episode of otitis media, 2 episodes of non-febrile gastroenteritis** |
| **18** | **1 episode of non-febrile upper airway infection** |
| **19** | **none** |
| **20** | **1 episode of febrile upper airway infection, 2 episodes of non-febrile upper airway infections, 1 follicular tonsillitis** |
| **21** | **1 episode of follicular tonsillitis** |
| **22** | **1 episode of non-febrile upper airway infection** |
| **23** | **1 episode of non-febrile upper airway infection** |
| **24** | **1 episode of non-febrile upper airway infection, 1 episode of non-febrile gastroenteritis** |
| **25** | **2 episodes of febrile upper airway infection, 1 gastroenteritis** |
| **26** | **1 episode of febrile upper airway infection** |
| **27** | **1 non-febrile gastroenteritis** |
| **28** | **1 episode of non-febrile upper airway infection** |
| **29** | **none** |
| **30** | **none** |
| **31** | **1 non-febrile gastroenteritis, 1 episode of follicular tonsillitis** |
| **32** | **1 episode of non-febrile upper airway infection** |
| **33** | **2 episodes of non-febrile upper airway infection** |
| **34** | **2 episodes of non-febrile upper airway infection** |
| **35** | **none** |
| **36** | **1 episode of non-febrile upper airway infection** |
| **37** | **1 episode of febrile upper airway infection** |
| **38** | **2 episodes of non-febrile upper airway infection** |
| **39** | **2 episodes of non-febrile upper airway infection, 1 follicular tonsillitis** |
| **40** | **1 gastroenteritis** |
| **41** | **2 episodes of non-febrile upper airway infection** |
| **42** | **2 episodes of non-febrile upper airway infection** |
| **43** | **1 episode of non-febrile upper airway infection, 2 gastroenteritis** |
| **44** | **1 episode of non-febrile upper airway infection** |
| **45** | **none** |

Supplementary Table 2.

TTV and CMV copy numbers of patients for each time-point who had positive CMV copy numbers during the study period. (TTV = log10 copy in plasma, CMV = copy number in plasma).

| **Pat. No.** | **TTV 1** | **CMV 1** | **TTV 2** | **CMV 2** | **TTV 3** | **CMV 3** | **TTV 4** | **CMV 4** | **TTV 5** | **CMV 5** | **TTV 6** | **CMV 6** | **TTV 7** | **CMV 7** | **TTV 8** | **CMV 8** | **TTV 9** | **CMV 9** | **TTV 10** | **CMV 10** | **TTV 11** | **CMV 11** | **TTV 12** | **CMV 12** |
| --- | --- | --- | --- | --- | --- | --- | --- | --- | --- | --- | --- | --- | --- | --- | --- | --- | --- | --- | --- | --- | --- | --- | --- | --- |
| **1** | 5,04 | neg | 6,00 | neg | 6,49 | neg | 6,70 | neg | 7,11 | 55 | 6,88 | neg | 6,58 | neg | 5,11 | neg | 6,40 | neg | 7,15 | neg | 7,15 | neg | 6,76 | neg |
| **2** | 8,68 | 186 | 7,94 | neg | 8,61 | 6260 | 8,00 | neg | 6,52 | neg | 7,98 | 100 | 7,61 | neg | 7,94 | neg | 7,71 | 28 | 7,59 | neg | 6,78 | neg | 7,60 | 34 |
| **3** | 6,20 | neg | 7,04 | neg | 6,59 | neg | 7,67 | neg | 5,75 | neg | 5,75 | neg | 5,46 | neg | 4,98 | neg | 4,58 | neg | 5,56 | neg | 5,23 | neg | 4,81 | neg |
| **4** | 5,04 | 138 | 6,08 | 115 | 4,23 | 39 | 5,46 | 83 | 5,70 | 126 | 6,30 | 22 | 7,86 | 336 | 8,34 | 1340 | 3,39 | neg | 2,85 | neg |  |  |  |  |
| **5** | 5,20 | neg | 5,54 | neg | 5,28 | neg | 5,48 | neg | 6,60 | neg | 4,86 | neg | 4,95 | neg | 5,15 | neg | 5,20 | neg | 5,18 | neg | 5,62 | neg | 5,62 | neg |
| **6** | 6,89 | neg | 6,53 | 34 | 6,92 | neg | 7,71 | neg | 6,83 | neg | 7,93 | neg | 7,97 | 76 | 7,48 | 70 | 8,69 | neg | 6,82 | neg | 7,81 | 24 | 6,48 | neg |
| **7** | 6,28 | neg | 6,23 | neg | 5,98 | neg | 6,04 | neg | 6,28 | neg | 6,54 | neg | 6,08 | neg | 5,79 | 56 | 4,66 | neg | 4,64 | neg | 5,73 | neg |  |  |
| **8** | 4,71 | neg | 5,36 | neg | 5,18 | neg | 6,32 | neg | 5,76 | neg | 5,77 | 108 | 5,18 | neg | 4,82 | neg | 4,46 | neg | 4,85 | neg | 5,15 | neg | 4,86 | neg |
